# SIRT6 Maintains Redox Homeostasis to Promote Porcine Oocyte Maturation

**DOI:** 10.3389/fcell.2021.625540

**Published:** 2021-02-25

**Authors:** Yu Li, Yilong Miao, Jingyue Chen, Bo Xiong

**Affiliations:** College of Animal Science and Technology, Nanjing Agricultural University, Nanjing, China

**Keywords:** SIRT6, oocyte maturation, meiotic failure, apoptosis, redox homeostasis

## Abstract

SIRT6, the sixth member of the sirtuin family proteins, has been characterized as a crucial regulator in multiple molecular pathways related to aging, including genome stability, DNA damage repair, telomere maintenance, and inflammation. However, the exact roles of SIRT6 during female germ cell development have not yet been fully determined. Here, we assessed the acquisition of meiotic competency of porcine oocytes by inhibition of SIRT6 activity. We observed that SIRT6 inhibition led to the oocyte meiotic defects by showing the impairment of polar body extrusion and cumulus cell expansion. Meanwhile, the compromised spindle/chromosome structure and actin dynamics were also present in SIRT6-inhibited oocytes. Moreover, SIRT6 inhibition resulted in the defective cytoplasmic maturation by displaying the disturbed distribution dynamics of cortical granules and their content ovastacin. Notably, we identified that transcript levels of genes related to oocyte meiosis, oxidative phosphorylation, and cellular senescence were remarkably altered in SIRT6-inhibited oocytes by transcriptome analysis and validated that the meiotic defects caused by SIRT6 inhibition might result from the excessive reactive oxygen species (ROS)-induced early apoptosis in oocytes. Taken together, our findings demonstrate that SIRT6 promotes the porcine oocyte meiotic maturation through maintaining the redox homeostasis.

## Introduction

During the meiotic maturation, mammalian fully grown oocytes at the prophase I stage undergo two key cellular events involving germinal vesicle breakdown and first polar body extrusion to reach the metaphase of the second meiosis (M II) stage awaiting for fertilization (Swann and Yu, 2008; Sun et al., 2009). Any errors that hinder the completion of nuclear or cytoplasmic maturation would prevent oocytes from acquiring the ability to fertilize and support the embryonic development, producing low-quality oocytes (Eppig, 1996; Holt et al., 2013). Previous studies have shown that the spindle disorganization, chromosome mis-segregation, mitochondrial dysfunction, and redox imbalance are often observed in low-quality oocytes, which are highly correlated with the occurrence of infertility, miscarriage, and congenital malformation (Eichenlaub-Ritter et al., 2004).

Sirtuin proteins (SIRT1–7) are a family of nicotinamide adenine dinucleotide (NAD^+^) enzymes that perform a diverse range of functions in the cells regarding energy metabolism, cellular stress resistance, genomic stability, and aging (Finkel et al., 2009). The founding member of the sirtuin protein family is yeast silent information regulator 2 (Sir2), which regulates the chromatin structure, DNA recombination, and gene expression (Vogt et al., 2008; Gao et al., 2009; Carafa et al., 2016; Watroba et al., 2017). In mammals, sirtuin proteins differ for the subcellular localization and functions (Morris, 2013; Pucci et al., 2013). Among them, SIRT6 acts as a nuclear deacetylase and ADP-ribosyltransferase that participates in the telomere function, metabolic homeostasis, DNA repair, and genome stability (Kanfi et al., 2012; Toiber et al., 2013; Tian et al., 2019). Under oxidative stress, SIRT6 is recruited to DNA double-strand breaks and associates with poly ADP-ribose polymerase 1 (PARP1) in the presence of DNA damage to activate the homologous and non-homologous end-joining recombination for damage repair (Morris, 2013; Pucci et al., 2013). However, the exact roles of SIRT6 in the female germ cells are not fully determined.

In the present study, we used porcine oocytes as a model to explore the function of SIRT6 on meiotic maturation, as the developmental and physiological indexes of porcine oocytes including the diameter of oocytes and maturation time are more similar to humans in comparison with mice (Schatten and Sun, 2009; Miao et al., 2017). We found that inhibition of SIRT6 activity impaired both nuclear and cytoplasmic maturation of oocytes, including the abnormal cytoskeleton assembly and cortical granule (CG) dynamics. We also discovered that SIRT6 inhibition compromised the transcript levels of genes related to oocyte meiosis, oxidative phosphorylation, and cellular senescence pathways by transcriptome analysis. The meiotic defects might be caused by the accumulated reactive oxygen species (ROS)-induced apoptosis.

## Materials and Methods

### Antibodies

Mouse monoclonal α-tubulin-fluorescein isothiocyanate (FITC) and acetyl-α-tubulin (Lys40) antibodies were obtained from Sigma-Aldrich (St. Louis, MO, United States; Cat# F2168, ABT241); rabbit monoclonal glyceraldehyde 3-phosphate dehydrogenase (GAPDH) and phospho-histone H2A.X (Ser139) antibodies were obtained from Cell Signaling Technology (Danvers, MA, United States; Cat# 2118, 9718); sheep polyclonal mouse BubR1 antibody was purchased from Abcam (Cambridge, MA, United States; Cat# ab28193); rabbit polyclonal human ovastacin antibody was obtained from Dr. Jurrien Dean lab (National Institutes of Health, Bethesda, MA, United States).

### Porcine Oocyte Collection and *in vitro* Maturation

Porcine ovaries were obtained from a local abattoir and transported to the laboratory in physiological saline containing streptomycin sulfate and penicillin G within 2 h after slaughtering. Cumulus cell–oocyte complexes (COCs) were aspirated from the follicles using a disposable syringe. COCs with compact cumulus cells were selected for *in vitro* maturation (IVM). The maturation medium is TCM-199 (Thermo Fisher Scientific, Waltham, MA, United States; Cat# 11150059) supplemented with 10% porcine follicular fluid, 5 μg/ml insulin, 10 ng/ml epidermal growth factor (EGF), 0.6 mM cysteine, 0.2 mM pyruvate, 25 μg/ml kanamycin, and 10 IU/ml of each equine chorionic gonadotropin (eCG) and human chorionic gonadotropin (hCG). Twenty germinal vesicle (GV) COCs were cultured in a drop of 100 μl maturation medium covered with mineral oil at 38.5°C, 5% CO_2_ for 26–28 h to metaphase I (M I) stage and for 42–44 h to M II stage.

### SIRT6-IN-1 Treatment

SIRT6-IN-1 (Selleckchem, Houston, TX, United States; Cat# S8627) was dissolved in dimethyl sulfoxide (DMSO) to 100 mM and diluted to a final concentration of 50 and 100 μM, respectively, with the maturation medium. The final concentration of DMSO in the maturation medium was not more than 0.1%.

### Fluorescence Staining and Confocal Microscopy

Denuded oocytes (DOs) were incubated in the fixation solution [4% paraformaldehyde/phosphate buffered saline (PBS)] for 30 min, in the permeabilization solution (1% Triton X-100/PBS) for 1 h, and in the blocking solution [1% bovine serum albumin (BSA)-supplemented PBS] for 1 h at room temperature (RT), followed by incubation with BubR1 antibody (1:100), α-tubulin-FITC antibody (1:200), acetyl-α-tubulin antibody (1:100), γH2A.X antibody (1:100), ovastacin antibody (1:100), phalloidin-TRITC (1:200; Sigma-Aldrich; Cat# P1951), or lens culinaris agglutinin (LCA)-FITC (1:200; Thermo Fisher Scientific; Cat# L32475) overnight at 4°C. After washes in phosphate buffered saline tween-20 (PBST), oocytes were incubated with the corresponding secondary antibodies for 1 h and counterstained with 10 μg/ml Hoechst 33342 or propidium iodide (PI) for 10 min at RT. In addition, oocytes were stained at 38.5°C for 30 min with 10 μM dichlorofluorescein diacetate (DCFHDA; Beyotime, Huangzhou, China; Cat# S0033S) for ROS staining and with Annexin-V-FITC (1:10; Beyotime, Huangzhou, China; Cat# C1062) for apoptosis assessment. Lastly, oocytes were mounted on the glass slides and imaged under a confocal microscope (LSM 700 META, Zeiss, Germany).

### Immunoblotting

A total of 100 porcine oocytes was collected in the lysis buffer (4 × LDS sample buffer, Thermo Fisher Scientific) with protease inhibitor and heated at 95°C for 5 min. Proteins were separated on 10% precast gels (Bis-Tris) and transferred to polyvinylidene fluoride (PVDF) membranes. The blots were then incubated in the blocking buffer [5% low-fat dry milk/tris buffered saline tween-20 (TBST)] for 1 h at RT and probed with acetyl-α-tubulin antibody (1:1,000) or GAPDH antibody (1:5,000) overnight at 4°C. After washes in TBST, the blots were incubated with the corresponding secondary antibodies for 1 h at RT. Chemiluminescence signals were acquired with ECL Plus (Thermo Fisher Scientific), and protein bands were detected by Tanon-3900 Imaging System.

### RNA Sequencing

Oocytes at M II stage were collected from control and SIRT6-inhibited groups (100 oocytes per group), and total RNA was extracted using RNeasy Micro Kit (Qiagen) according to manufacturer’s instructions. Extracted RNA was quantified with the Qubit RNA Assay Kit (Thermo Fisher Scientific). mRNA library construction was performed with NEBNext Ultra RNA Library Prep Kit for Illumina (New England Biolabs) according to the manuals. The protocol consisted of sequential RNA fragmentation, reverse transcription using random primers, second strand cDNA synthesis, end repair, dA-tailing, adapter ligation, and PCR enrichment. The concentration and quality of libraries were tested by a NanoDrop 2000 spectrophotometer (Thermo Fisher Scientific), qPCR, and Agilent 2100 Bioanalyzer (Agilent Technologies, Palo Alto, CA, United States). Then, the libraries were sequenced on Illumina Hiseq X Ten instruments with 150-bp pair-end reads. All clusters that passed the quality filter were exported into fastq files.

### RNA Isolation and Quantitative Real-Time PCR

Total RNA was extracted from a total of 30 oocytes using RNeasy Mini Kit (Qiagen, Germantown, MD, United States) and reversed to cDNA using PrimeScript RT Master Mix (Takara, Kusatsu, Shiga, Japan), followed by storing at −20°C until use. Quantitative real-time PCR was conducted using SYBR Green PCR Master Mix with QuantStudio 7 Flex Real-Time PCR System (Thermo Fisher, Waltham, MA, United States). Data were normalized against GAPDH, and quantification of the fold change was determined by the comparative CT method. The primers were listed as follows:

*FOXO1* (F: CGCCACCATACCTATCGTCC/R: GGGAAT ACGTGTGCCCAGAA);

*PPP1CC* (F: TCTATGGAGCAGATTCGGCG/R:CGGGGT CAGACCACAAAAGA);

*NFATC2* (F: CACCTGGGGTGTTGGTTTGA/R: CTGCAG CTTCGCAGAAATCC);

*PNN* (F: CCATCCAAGCCAGATTGCTG/R: GATTCTC TCCGAGTCCGACG);

*SRSF10* (F: GACCGACTGGAAGACCACG/R: GTAGCAG CTTACAGGGCCAA);

*GAPDH*: (F: GATGGTGAAGGTCGGAGTG/R: CGAAGTT GTCATGGATGACC).

### Statistical Analysis

All percentages or values from at least three repeated experiments were expressed as the mean ± SEM or mean ± SD, and the number of oocytes observed was labeled in parentheses as (*n*). Data were analyzed by one-way ANOVA or *t*-test, provided by GraphPad Prism 8 statistical software. The level of significance was accepted as *P* < 0.05.

## Results

### Inhibition of SIRT6 Perturbs the Porcine Oocyte Meiotic Maturation and Activates Spindle Assembly Checkpoint

**FIGURE 1:**
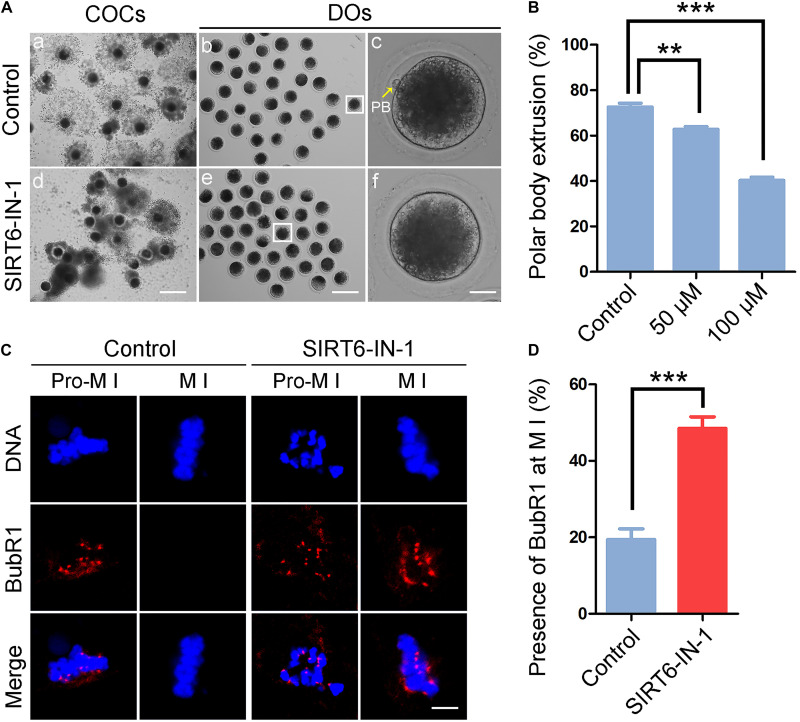
Effect of SIRT6 inhibition on meiotic maturation and spindle assembly checkpoint (SAC) activation in porcine oocytes. **(A)** Representative images of *in vitro*-matured oocytes in control and SIRT6-inhibited groups. Polar body extrusion (PBE) of denuded oocytes (DOs) and cumulus cell expansion of cumulus cell–oocyte complexes (COCs) were imaged by the confocal microscope. Scale bar, 360 μm **(a,d)**; 240 μm **(b,e)**; 30 μm **(c,f)**. **(B)** The proportion of PBE was calculated in control and different concentrations of SIRT6-inhibited groups (50 and 100 μM) after 44 h *in vitro* culture. **(C)** The localization of BubR1 on the chromosomes in control and SIRT6-inhibited oocytes at pro-metaphase I (M I) and M I stages. Porcine oocytes were immunostained with BubR1 antibody and counterstained with Hoechst. Scale bar, 10 μm. **(D)** The proportion of BubR1 presence at the M I stage was calculated in control and SIRT6-inhibited oocytes. Data of panels **(B,D)** were shown as the mean percentage (mean ± SEM) of at least three independent experiments. **P < 0.01, ***P < 0.001.

To examine the function of SIRT6 during the porcine oocyte meiotic maturation, a cell-permeable SIRT6-specific inhibitor, SIRT6-IN-1, was supplemented to the *in vitro* maturation medium (50 or 100 μM) to assess the developmental status of COCs. As displayed in Figure 1A, most of the cumulus cells surrounding control oocytes were well expanded after *in vitro* maturation for 44 h, while those surrounding SIRT6-inhibited oocytes were only expanded partially or even not expanded entirely (Figure 1A). Furthermore, a majority of oocytes developed to M II stage with the first polar body in the control group but failed after inhibition of SIRT6 (Figure 1A). Quantitative results indicated that the rate of polar body extrusion (PBE) was decreased in a dose-dependent manner in SIRT6-inhibited oocytes in comparison with the controls (control: 72.6% ± 1.7%, *n* = 127; 50 μM: 62.8% ± 1.1%, *n* = 129, *P* < 0.01; 100 μM: 40.3% ± 1.4%, *n* = 129, *P* < 0.001; Figure 1B). We finally used the concentration of 100 μM SIRT6-IN-1 for subsequent studies.

A high frequency of meiotic arrest in SIRT6-inhibited oocytes implies that spindle assembly checkpoint (SAC) might be provoked. To test it, oocytes were immunostained for BubR1, a pivotal component of SAC, to indicate the SAC activity. In control oocytes, BubR1 located to the kinetochores at pro-M I stage and released to the cytoplasm at M I stage (Figure 1C). However, BubR1 was still present at kinetochores at M I stage in SIRT6-inhibited oocytes, indicative of persistent activation of SAC (19.5% ± 2.8%, *n* = 36 vs. 48.5% ± 3.0%, *n* = 33, *P* < 0.001; Figures 1C,D).

### Inhibition of SIRT6 Impairs the Spindle/Chromosome Structure in Porcine Oocytes

**FIGURE 2:**
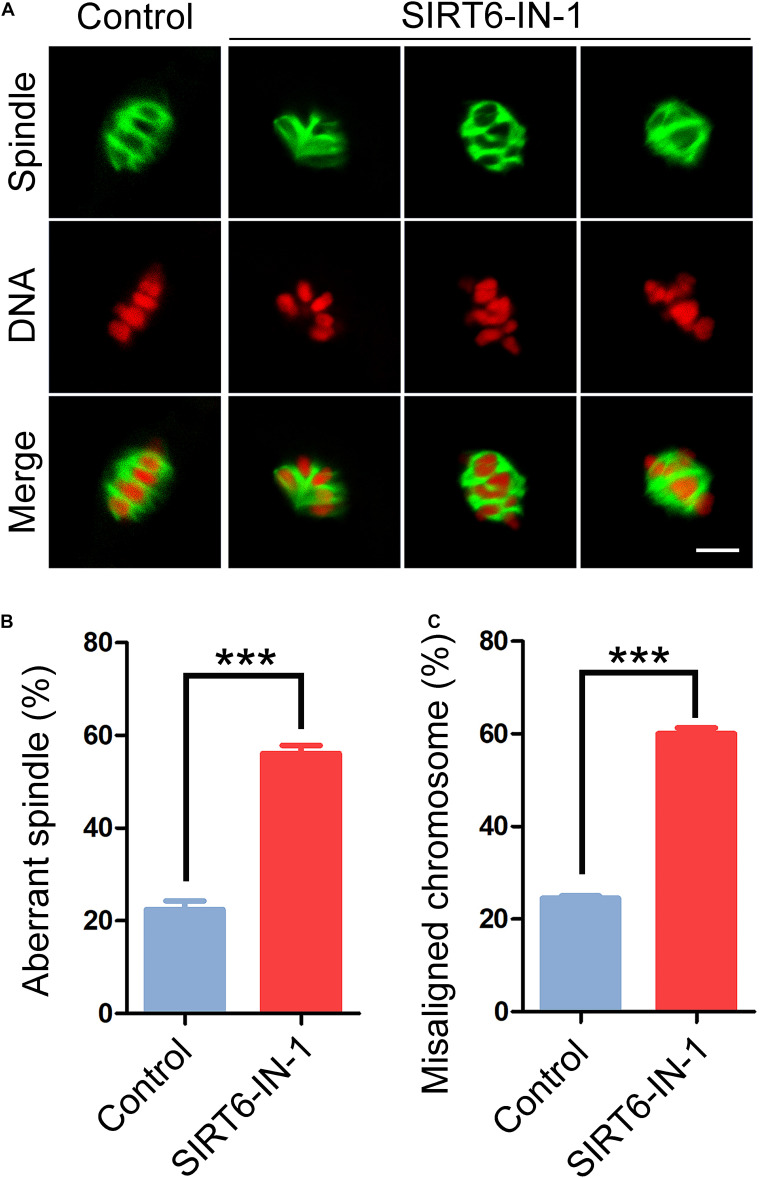
Effect of SIRT6 inhibition on the spindle assembly and chromosome alignment in porcine oocytes. **(A)** Representative images of spindle/chromosome structure in control and SIRT6-inhibited oocytes. Porcine oocytes were immunostained with α-tubulin-FITC antibody to show the spindle morphologies and were counterstained with propidium iodide (PI) to display the chromosome alignment. Scale bar, 10 μm. **(B)** The proportion of abnormal spindles was calculated in control and SIRT6-inhibited oocytes. **(C)** The proportion of misaligned chromosomes was calculated in control and SIRT6-inhibited oocytes. Data of panels **(B,C)** were expressed as the mean percentage (mean ± SEM) of at least three independent experiments. ***P < 0.001.

To ask whether the persistent activation of SAC observed in SIRT6-inhibited oocytes results from the defects in spindle assembly and chromosome alignment, M I oocytes were immunostained with α-tubulin antibody to show the spindle morphology and counterstained with PI to exhibit the chromosome alignment. The immunofluorescent images indicated that control oocytes displayed a typical barrel-shaped spindle apparatus with well-aligned chromosomes at the equatorial plate (Figure 2A). On the contrary, SIRT6-inhibited oocytes presented a much higher incidence of aberrant spindles and misaligned chromosomes (spindle: 22.4% ± 1.9%, *n* = 49 vs. 56.0% ± 1.7%, *n* = 47, *P* < 0.001; chromosome: 24.5% ± 0.5%, *n* = 49 vs. 60.1% ± 1.2%, *n* = 50, *P* < 0.001; Figures 2A–C), suggesting that SIRT6 inhibition results in the defective spindle/chromosome structure, which is a major cause for the SAC activation and oocyte meiotic arrest.

### Inhibition of SIRT6 Reduces the Level of Acetylated α-Tubulin in Porcine Oocytes

**FIGURE 3:**
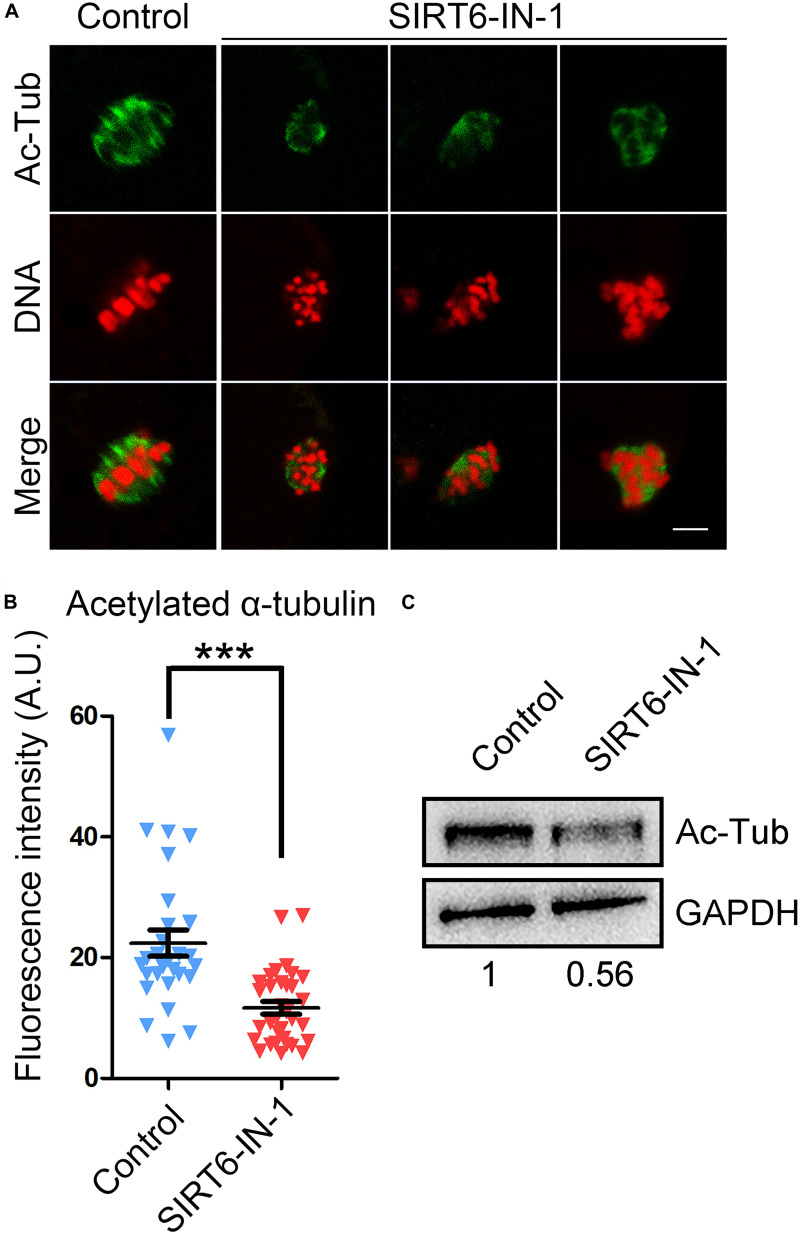
Effect of SIRT6 inhibition on the level of acetylated α-tubulin in porcine oocytes. **(A)** Representative images of α-tubulin acetylation in control and SIRT6-inhibited oocytes. Porcine oocytes were immunostained with acetyl-α-tubulin (Lys40) antibody and counterstained with propidium iodide (PI). Scale bar, 10 μm. **(B)** The fluorescence intensity of acetyl-α-tubulin signals was quantified in control and SIRT6-inhibited oocytes. Data were expressed as the mean percentage (mean ± SEM) of at least three independent experiments. ***P < 0.001. **(C)** The levels of acetylated α-tubulin in control and SIRT6-inhibited oocytes were detected by immunoblotting. The oocytes were immunoblotted for acetyl-α-tubulin (Lys40) and glyceraldehyde 3-phosphate dehydrogenase (GAPDH), respectively.

Since normal spindle organization is dependent on the microtubule dynamics, we then evaluated the microtubule stability by assessing the acetylation level of α-tubulin, a sign of the stable microtubules that has been validated in mammalian oocytes. We found that SIRT6 inhibition remarkably reduced the signals of acetyl-α-tubulin in comparison with the controls, as assessed by the fluorescence imaging and intensity measurement (11.7 ± 1.1, *n* = 33 vs. 22.4 ± 2.2, *n* = 28, *P* < 0.001; Figures 3A,B), which was further verified by the immunoblotting analysis (Figure 3C). These data indicate that SIRT6 inhibition produces the less stable microtubules and thus disrupts spindle organization.

### Inhibition of SIRT6 Disturbs the Actin Cytoskeleton in Porcine Oocytes

**FIGURE 4:**
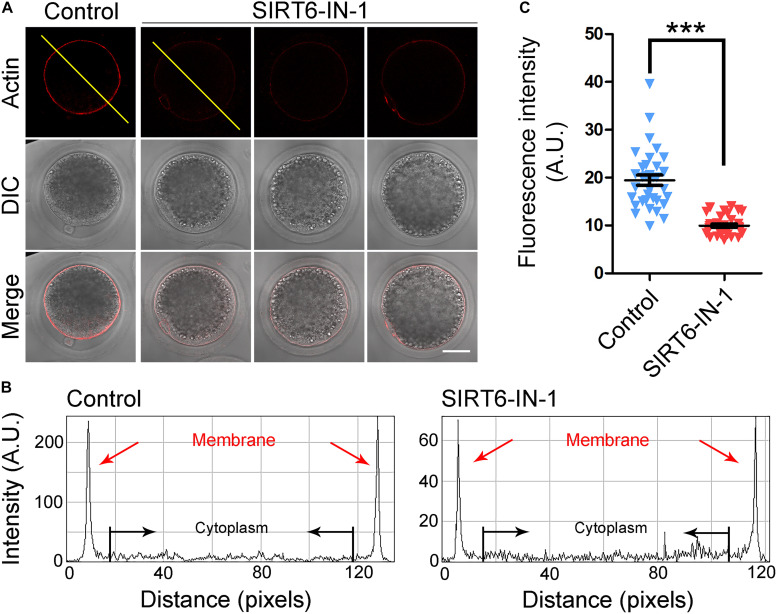
Effect of SIRT6 inhibition on the actin dynamics in porcine oocytes. **(A)** Representative images of actin filaments in control and SIRT6-inhibited oocytes. Porcine oocytes were stained with phalloidin-TRITC to show the actin filaments. Scale bar, 40 μm. **(B)** The graphs showed the fluorescence intensity profiling of actin filaments in control and SIRT6-inhibited oocytes. Pixel intensities were measured along the lines drawn across the oocytes. **(C)** The fluorescence intensity of actin signals was quantified in control and SIRT6-inhibited oocytes. Data were expressed as the mean percentage (mean ± SEM) of at least three independent experiments. ***P < 0.001.

In meiosis, the actin filaments take a critical part in chromosome movement, spindle positioning, and cortical polarization during oocyte maturation (Azoury et al., 2008; Field and Lenart, 2011; Duan and Sun, 2019). To further investigate whether SIRT6 drives oocyte meiosis by affecting the actin cytoskeleton, phalloidin-TRITC was used to label the F-actin for displaying the actin filaments. As presented in Figure 4A, actin filaments were identically concentrated on the plasma membrane with strong signals in control oocytes. By striking contrast, SIRT6-inhibited oocytes showed the impaired assembly of actin filaments with weak signals (Figure 4A), which was verified by the fluorescence plot profiling that was measured along the lines drawn across the oocytes (Figure 4B). In addition, the quantification of fluorescence intensity on the plasma membrane revealed that the signals of actin filaments were significantly decreased in SIRT6-inhibited oocytes (19.5 ± 1.1, *n* = 34 vs. 10.0 ± 0.3, *n* = 34, *P* < 0.01; Figure 4C), implying that SIRT6 is implicated in the actin dynamics to drive the porcine oocyte meiotic maturation.

### SIRT6 Is Essential for the Correct Distribution of Cortical Granules and Ovastacin in Porcine Oocytes

**FIGURE 5:**
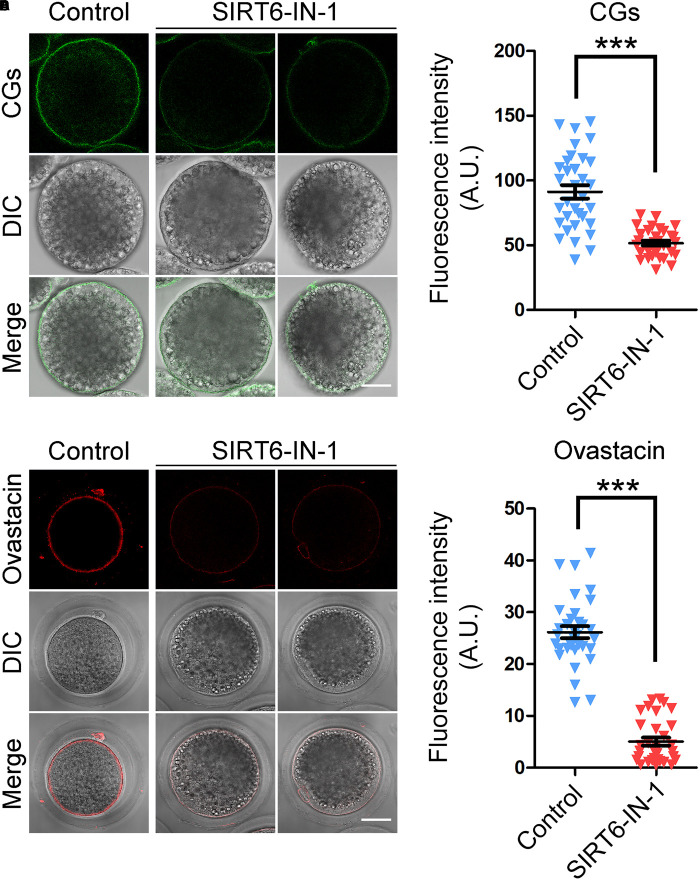
Effect of SIRT6 inhibition on the localization patterns of cortical granules (CGs) and ovastacin in porcine oocytes. **(A)** Representative images of CG distribution in control and SIRT6-inhibited oocytes. Porcine oocytes were stained with LCA-FITC to display the CGs. Scale bar, 30 μm. **(B)** The fluorescence intensity of LCA signals was quantified in control and SIRT6-inhibited oocytes. **(C)** Representative images of ovastacin distribution in control and SIRT6-inhibited oocytes. Porcine oocytes were immunostained with ovastacin antibody and imaged by confocal microscope. Scale bar, 30 μm. **(D)** The fluorescence intensity of ovastacin signals was quantified in control and SIRT6-inhibited oocytes. Data of panels **(B,D)** were expressed as the mean percentage (mean ± SEM) of at least three independent experiments. ***P < 0.001.

The distribution of CGs, which are the oocyte-specific vesicles implicated in the post-fertilization block to polyspermy (Sun et al., 2001; Burkart et al., 2012), is regarded as one of the key indexes of oocyte cytoplasmic maturation. Therefore, we labeled the CGs with FITC-conjugated LCA to visualize their distribution dynamics. As displayed in Figure 5A, CGs distributed in the subcortex of oocytes with uniform and robust signals. Whereas they lost this regular localization pattern in SIRT6-inhibited oocytes by showing incontinuous and faded signals (Figure 5A). The quantitation result of fluorescence intensity revealed that CG signals had a substantial decrease in SIRT6-inhibited oocytes compared to the controls (91.3 ± 5.1, *n* = 33 vs. 51.6 ± 1.9, *n* = 32, *P* < 0.001; Figure 5B).

Furthermore, we detected the distribution of ovastacin, a content of CGs that regulates the post-fertilization sperm binding to prevent polyspermy. If ovastacin is released out of oocytes before fertilization, it would lead to the premature loss of sperm-binding sites in the zona pellucida (ZP) of oocytes. Consistent with the distribution pattern of CGs, we found that ovastacin was localized in the subcortical region with strong signals in control oocytes. On the contrary, the much lower signals of ovastacin were observed in SIRT6-inhibited oocytes (Figure 5C). As expected, the fluorescence intensity quantification revealed that ovastacin signals were considerably decreased in SIRT6-inhibited oocytes than those in controls (5.0 ± 0.8, *n* = 32 vs. 26.2 ± 1.1, *n* = 43, *P* < 0.001; Figure 5D), indicating that SIRT6 inhibition compromises the cytoplasmic maturation of porcine oocytes.

### Transcriptome Analysis Identifies Target Effectors of SIRT6 in Porcine Oocytes

**FIGURE 6:**
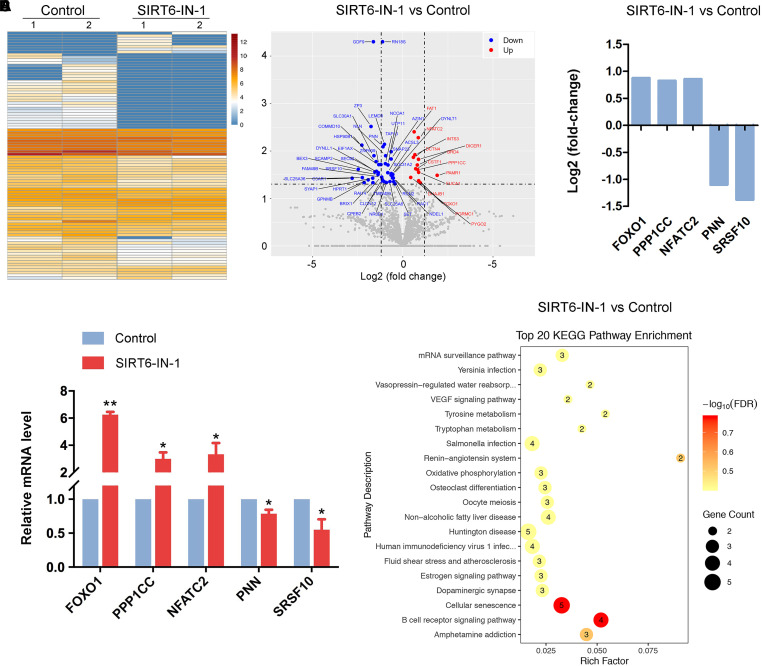
Effect of SIRT6 inhibition on the transcriptome profile of porcine oocytes. **(A)** Heatmap illustration displayed the differentially expressed genes (DEGs) between control and SIRT6-inhibited oocytes. **(B)** Volcano plot showed the DEGs in SIRT6-inhibited oocytes compared to controls. Downregulated, blue; upregulated, red. **(C)** RNA sequencing (RNA-seq) results of selected genes in SIRT6-inhibited oocytes compared to controls. **(D)** Validation of RNA-seq data by quantitative RT-PCR in control (blue) and SIRT6-inhibited (red) oocytes. Data were presented as the mean percentage (mean ± SEM) of at least three independent experiments. **P < 0.01, *P < 0.05. **(E)** Kyoto Encyclopedia of Genes and Genomes (KEGG) analysis of DEGs in SIRT6-inhibited oocytes in comparison with controls.

To gain insight into the exact roles of SIRT6 during porcine oocyte maturation, we profiled the transcriptome of M II oocytes from control and SIRT6-inhibited groups by RNA sequencing to identify the potential downstream effectors. Heatmap and volcano plot data indicated that 94 differentially expressed genes (DEGs) were identified in SIRT6-inhibited oocytes compared to the controls (Figures 6A,B). Among them, 71 DEGs were downregulated and 23 DEGs were upregulated (Figure 6B). Transcript levels of several randomly selected genes were verified by quantitative real-time PCR (Figures 6C,D). In particular, Kyoto Encyclopedia of Genes and Genomes (KEGG) enrichment analysis showed that DEGs were highly enriched in the pathways related to oocyte meiosis, oxidative phosphorylation, and cellular senescence (Figure 6E). The oocyte meiosis pathway confirmed our above observations about meiotic defects in SIRT6-inhibited oocytes. The pathways of oxidative phosphorylation and cellular senescence indicated that dysfunction of SIRT6 might induce oxidative stress and apoptosis in porcine oocytes.

### Inhibition of SIRT6 Produces Excessive Reactive Oxygen Species, Accumulated DNA Damage, and Apoptosis in Porcine Oocytes

**FIGURE 7:**
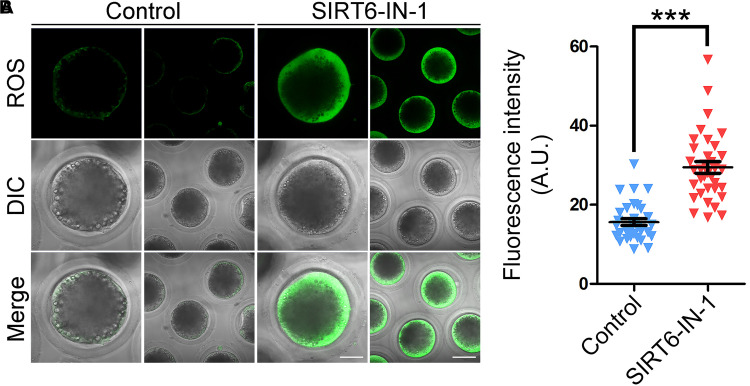
Effect of SIRT6 inhibition on the reactive oxygen species (ROS) levels in porcine oocytes. **(A)** Representative images of ROS levels by DCFH staining in control and SIRT6-inhibited oocytes. Scale bars, 40 and 80 μm. **(B)** The fluorescence intensity of ROS signals was quantified in control and SIRT6-inhibited oocytes. Data were expressed as the mean percentage (mean ± SEM) of at least three independent experiments. ***P < 0.001.

In line with our transcriptome data, it has been reported that SIRT6 plays a pivotal role in reducing oxidative stress and suppressing apoptosis in mitotic cells (Fan et al., 2019). Hence, we investigated whether this is the case in porcine oocytes. For this purpose, ROS levels were measured by DCFH staining. As presented in Figure 7A, ROS signals were hardly observed in control oocytes. However, SIRT6 inhibition prominently elevated the signal levels in the oocyte cytoplasm (Figure 7A). Accordingly, the fluorescence intensity of ROS signals was much higher in SIRT6-inhibited oocytes compared to the controls (29.5 ± 1.5, *n* = 35 vs. 15.6 ± 0.9, *n* = 32, *P* < 0.001; Figure 7B).

**FIGURE 8:**
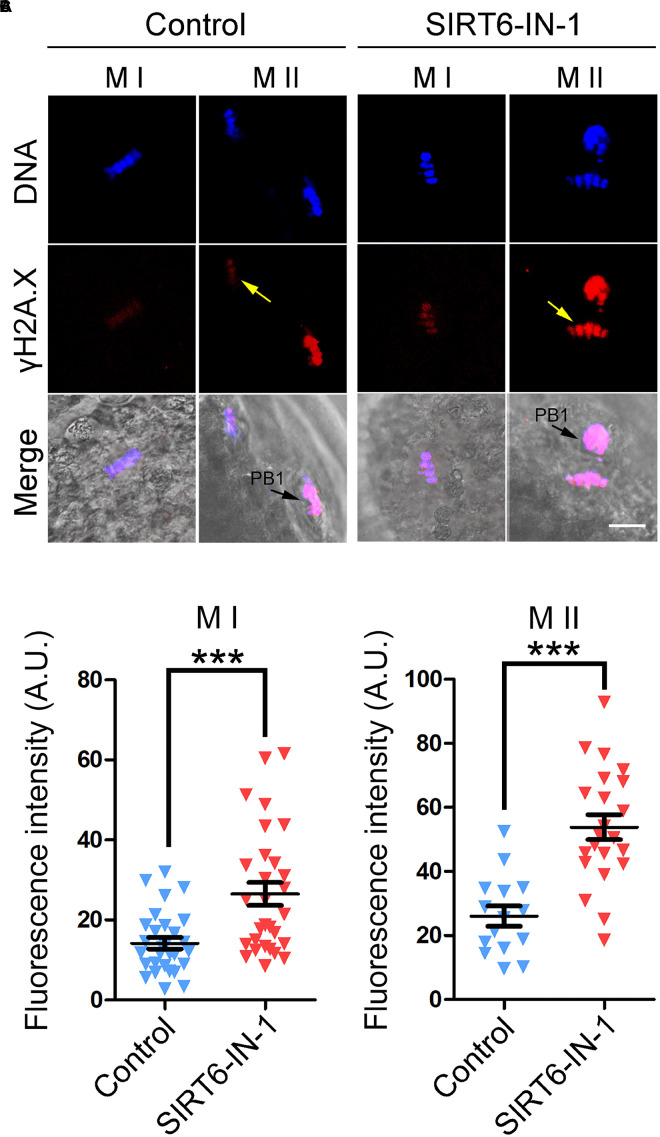
Effect of SIRT6 inhibition on the levels of DNA damage in porcine oocytes. **(A)** Representative images of γH2A.X accumulation in control and SIRT6-inhibited oocytes. Porcine oocytes were immunostained with γH2A.X antibody and imaged by confocal microscope. Scale bar, 20 μm. PB1, first polar body. **(B)** The fluorescence intensity of γH2A.X signals was quantified in control and SIRT6-inhibited oocytes at the metaphase I (M I) stage. **(C)** The fluorescence intensity of γH2A.X signals was quantified in control and SIRT6-inhibited oocytes at the M II stage. Data of panels **(B,C)** were expressed as the mean percentage (mean ± SEM) of at least three independent experiments. ***P < 0.001.

Because excessive ROS usually leads to DNA damage accumulation and SIRT6 plays an important role in DNA damage repair, including DNA double-strand break repair and base excision repair (Mao et al., 2011; Rizzo et al., 2017; Tian et al., 2019), we then examined the level of DNA damage after SIRT6 inhibition by γH2A.X antibody staining in porcine oocytes. The fluorescent imaging and intensity measurement displayed that γH2A.X signals dramatically accumulated in SIRT6-inhibited oocytes compared to the controls at both M I stage (14.2 ± 1.4, *n* = 29 vs. 26.5 ± 2.9, *n* = 29, *P* < 0.001; Figures 8A,B) and M II stage (26.0 ± 1.4, *n* = 15 vs. 53.8 ± 3.9, *n* = 22, *P* < 0.001; Figures 8A,C), suggesting that the DNA damage repair is compromised in SIRT6-inhibited oocytes.

**FIGURE 9:**
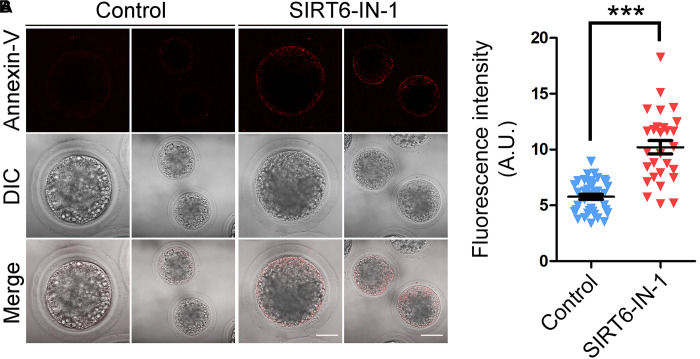
Effect of SIRT6 inhibition on the early apoptosis in porcine oocytes. **(A)** Representative images of apoptotic oocytes by Annexin-V staining in control and SIRT6-inhibited groups. Scale bars, 40 and 80 μm. **(B)** The proportion of apoptotic oocytes was calculated in control and SIRT6-inhibited groups. Data were expressed as the mean percentage (mean ± SEM) of at least three independent experiments. ***P < 0.001.

We next examined the apoptotic status of SIRT6-inhibited oocytes by Annexin-V staining. The imaging result showed that Annexin-V signals were hardly detected in control oocytes but evidently present on the membrane of SIRT6-inhibited oocytes (Figure 9A). Also, the proportion of apoptosis was significantly higher in SIRT6-inhibited oocytes than that in controls (5.8 ± 0.2, *n* = 39 vs. 10.2 ± 0.6, *n* = 28, *P* < 0.001; Figure 9B). Overall, these observations demonstrate that SIRT6 inhibition produces excessive ROS to induce apoptosis in porcine oocytes.

## Discussion

Sir2 in yeast is a heterochromatin molecule that functions in gene silencing and longevity (Imai et al., 2000). In mammals, seven homologs (sirtuins 1–7) of Sir2 have been characterized, varying in tissue specificity, cellular localization, enzymatic activity, and targets (Houtkooper et al., 2012). Many studies have demonstrated that sirtuins are implicated in the regulation of cellular metabolism, aging, and apoptosis (Houtkooper et al., 2012). SIRT6, as the distant mammalian Sir2 homolog, is a nuclear protein that has been involved in various biological processes, such as metabolic homeostasis, DNA damage repair, inflammation, aging, and tumorigenesis (Finkel et al., 2009). Previous study has revealed that SIRT6 is essential for the proper assembly of meiotic apparatus during mouse oocyte development (Han et al., 2015). However, whether this function of SIRT6 in meiosis is conserved among species and the potential underlying mechanisms are still unclear.

To address this question, we applied porcine oocytes as a research model, as they have the developmental and physiological similarities with humans in comparison with mice. We firstly investigated the effect of SIRT6 inhibition on polar body extrusion and cumulus cell expansion, the key indicators for oocyte maturation. By treating with SIRT6-IN-1, the oocytes exhibited a dramatically decreased incidence of polar body extrusion with poor expansion of cumulus cells, indicating that SIRT6 is indispensable for the proper porcine oocyte meiotic maturation. The high occurrence of meiotic arrest predicts that SAC might be persistently activated at the M I stage. As expected, our findings showed that SIRT6 inhibition resulted in various spindle/chromosome abnormalities that resultantly provoked the SAC. Consistently, a recent study displayed that treatment of porcine COCs by the same SIRT6 inhibitor also led to a remarkable reduction of polar body extrusion and impairment of spindle organization and chromosome alignment (Cao et al., 2020). Additionally, our data validated that inhibition of SIRT6 perturbed the microtubule dynamics by showing a lowered level of acetylated α-tubulin, an index of stabilized microtubules, suggesting that the impairment of microtubule stability might be a major cause leading to abnormal spindle organization.

As an essential component of the cytoskeleton, actin filaments are known to participate in spindle positioning, intracellular transport, and asymmetric division in mammalian oocytes (Field and Lenart, 2011). Our data illustrated that significant reduction of actin signals on the plasma membrane was observed after SIRT6 inhibition, suggesting that the impaired actin dynamics may be another reason resulting in oocyte meiotic failure when SIRT6 is inhibited.

Mammalian CGs are oocyte-specific vesicles that locate in the subcortical region of fully grown oocytes to function in the post-fertilization block to polyspermy (Burkart et al., 2012). The correct distribution of CGs is usually considered an indicator of oocyte cytoplasmic maturation. Our observations revealed that the distribution pattern of CGs was perturbed in the subcortex of SIRT6-inhibited oocytes, suggesting that cytoplasmic maturation is hindered. Ovastacin, a content of CGs, cleaves N-terminal domain of ZP2 following fertilization to prevent sperm binding to the ZP surrounding fertilized oocytes (Burkart et al., 2012). In agreement with the disturbance of CG dynamics, the distribution of ovastacin was also perturbed in SIRT6-inhibited oocytes, implying that their sperm binding and fertilization ability would be affected.

Lastly, our findings illustrated that transcript levels of genes related to oxidative stress and apoptosis were dramatically altered, as assessed by transcriptome analysis. Although the transcription activity is almost quiescent during oocyte meiotic maturation, SIRT6 might indirectly modulate the mRNA stability of related genes instead of their expression. It has been reported that SIRT6 has a role in the regulation of cyclooxygenase (COX)-2 mRNA stability by repressing AMP-activated protein kinase (AMPK) signaling pathway in human skin squamous cell carcinoma (Ming et al., 2014). Concordantly, elevated levels of ROS were observed in SIRT6-inhibited oocytes, which would attack the macromolecules in the cells such as DNA and thus cause DNA damage accumulation, consequently leading to the generation of early apoptosis. This might be the main mechanism for the oocyte meiotic failure.

## Conclusion

We evidence that SIRT6 performs an indispensable function to drive porcine oocyte meiotic maturation through maintaining meiotic organelle dynamics, including spindle/chromosome structure formation, actin polymerization, and CG distribution. Meanwhile, we discover that these meiotic defects might be caused by excessive ROS-induced occurrence of DNA damage and apoptosis. Our study extends the understanding about the molecular basis underlying oocyte development arrest that frequently occurs during *in vitro* maturation.

## Data Availability Statement

The datasets generated for this study can be found in online repositories. The names of the repository/repositories and accession number(s) can be found below: https://www.ncbi.nlm.nih.gov/geo/, GSE161068.

## Ethics Statement

The animal study was reviewed and approved by the Animal Research Institute Committee of Nanjing Agricultural University, China.

## Author Contributions

BX designed the research. YL, YM, and JC performed the experiments. YL and BX analyzed the data. YL and BX wrote the manuscript. All authors contributed to the article and approved the submitted version.

## Conflict of Interest

The authors declare that the research was conducted in the absence of any commercial or financial relationships that could be construed as a potential conflict of interest.

## References

[B1] Azoury J. Lee K. W. Georget V. Rassinier P. Leader B. Verlhac M. H. (2008). Spindle positioning in mouse oocytes relies on a dynamic meshwork of actin filaments. *Curr. Biol.* 18 1514–1519. 10.1016/j.cub.2008.08.044 18848445

[B2] Burkart A. D. Xiong B. Baibakov B. Jimenez-Movilla M. Dean J. (2012). Ovastacin, a cortical granule protease, cleaves ZP2 in the zona pellucida to prevent polyspermy. *J. Cell Biol.* 197 37–44. 10.1083/jcb.201112094 22472438 PMC3317803

[B3] Cao Z. Zhang D. Tong X. Wang Y. Qi X. Ning W. (2020). Cumulus cell-derived and maternal SIRT6 differentially regulates porcine oocyte meiotic maturation. *Theriogenology* 142 158–168. 10.1016/j.theriogenology.2019.09.048 31593883

[B4] Carafa V. Rotili D. Forgione M. Cuomo F. Serretiello E. Hailu G. S. (2016). Sirtuin functions and modulation: from chemistry to the clinic. *Clin. Epigenet.* 8:61. 10.1186/s13148-016-0224-223 PMC4879741 27226812

[B5] Duan X. Sun S. C. (2019). Actin cytoskeleton dynamics in mammalian oocyte meiosis. *Biol. Reprod.* 100 15–24. 10.1093/biolre/ioy163 30010726

[B6] Eichenlaub-Ritter U. Vogt E. Yin H. Gosden R. (2004). Spindles, mitochondria and redox potential in ageing oocytes. *Reprod. Biomed. Online* 8 45–58. 10.1016/s1472-6483(10)60497-x 14759287

[B7] Eppig J. J. (1996). Coordination of nuclear and cytoplasmic oocyte maturation in eutherian mammals. *Reprod. Fertil. Dev.* 8 485–489. 10.1071/rd9960485 8870074

[B8] Fan Y. Yang Q. Yang Y. Gao Z. Ma Y. Zhang L. (2019). Sirt6 suppresses high glucose-induced mitochondrial dysfunction and apoptosis in podocytes through AMPK activation. *Int. J. Biol. Sci.* 15 701–713. 10.7150/ijbs.29323 30745856 PMC6367578

[B9] Field C. M. Lenart P. (2011). Bulk cytoplasmic actin and its functions in meiosis and mitosis. *Curr. Biol.* 21 R825–R830. 10.1016/j.cub.2011.07.043 21996509

[B10] Finkel T. Deng C. X. Mostoslavsky R. (2009). Recent progress in the biology and physiology of sirtuins. *Nature* 460 587–591. 10.1038/nature08197 19641587 PMC3727385

[B11] Gao G. Wesolowska N. Rong Y. S. (2009). SIRT combines homologous recombination, site-specific integration, and bacterial recombineering for targeted mutagenesis in *Drosophila*. *Cold Spring Harb. Protoc.* 2009:pdb.prot5236. 10.1101/pdb.prot5236 20147194

[B12] Han L. Ge J. Zhang L. Ma R. Hou X. Li B. (2015). Sirt6 depletion causes spindle defects and chromosome misalignment during meiosis of mouse oocyte. *Sci. Rep.* 5:15366. 10.1038/srep15366 26481302 PMC4612726

[B13] Holt J. E. Lane S. I. Jones K. T. (2013). The control of meiotic maturation in mammalian oocytes. *Curr. Top Dev. Biol.* 102 207–226. 10.1016/B978-0-12-416024-8.00007-6 23287034

[B14] Houtkooper R. H. Pirinen E. Auwerx J. (2012). Sirtuins as regulators of metabolism and healthspan. *Nat. Rev. Mol. Cell Biol.* 13 225–238. 10.1038/nrm3293 22395773 PMC4872805

[B15] Imai S. Armstrong C. M. Kaeberlein M. Guarente L. (2000). Transcriptional silencing and longevity protein Sir2 is an NAD-dependent histone deacetylase. *Nature* 403 795–800. 10.1038/35001622 10693811

[B16] Kanfi Y. Naiman S. Amir G. Peshti V. Zinman G. Nahum L. (2012). The sirtuin SIRT6 regulates lifespan in male mice. *Nature* 483 218–221. 10.1038/nature10815 22367546

[B17] Mao Z. Hine C. Tian X. Van Meter M. Au M. Vaidya A. (2011). SIRT6 promotes DNA repair under stress by activating PARP1. *Science* 332 1443–1446. 10.1126/science.1202723 21680843 PMC5472447

[B18] Miao Y. Zhou C. Cui Z. Tang L. Shiyang X. Lu Y. (2017). Dynein promotes porcine oocyte meiotic progression by maintaining cytoskeletal structures and cortical granule arrangement. *Cell Cycle* 16 2139–2145. 10.1080/15384101.2017.1380133 28933593 PMC5731405

[B19] Ming M. Han W. Zhao B. Sundaresan N. R. Deng C. X. Gupta M. P. (2014). SIRT6 promotes COX-2 expression and acts as an oncogene in skin cancer. *Cancer Res.* 74 5925–5933. 10.1158/0008-5472.CAN-14-1308 25320180 PMC4203414

[B20] Morris B. J. (2013). Seven sirtuins for seven deadly diseases of aging. *Free Radic Biol. Med.* 56 133–171. 10.1016/j.freeradbiomed.2012.10.525 23104101

[B21] Pucci B. Villanova L. Sansone L. Pellegrini L. Tafani M. Carpi A. (2013). Sirtuins: the molecular basis of beneficial effects of physical activity. *Int. Emerg. Med.* 8(Suppl. 1), S23–S25. 10.1007/s11739-013-0920-3 23462891

[B22] Rizzo A. Iachettini S. Salvati E. Zizza P. Maresca C. D’angelo C. (2017). SIRT6 interacts with TRF2 and promotes its degradation in response to DNA damage. *Nucleic Acids Res.* 45 1820–1834. 10.1093/nar/gkw1202 27923994 PMC5389694

[B23] Schatten H. Sun Q. Y. (2009). The functional significance of centrosomes in mammalian meiosis, fertilization, development, nuclear transfer, and stem cell differentiation. *Environ. Mol. Mutagen.* 50 620–636. 10.1002/em.20493 19402157

[B24] Sun Q. Y. Miao Y. L. Schatten H. (2009). Towards a new understanding on the regulation of mammalian oocyte meiosis resumption. *Cell Cycle* 8 2741–2747. 10.4161/cc.8.17.9471 19717979

[B25] Sun Q. Y. Wu G. M. Lai L. Park K. W. Cabot R. Cheong H. T. (2001). Translocation of active mitochondria during pig oocyte maturation, fertilization and early embryo development in vitro. *Reproduction* 122 155–163. 10.1530/reprod/122.1.155 11425340

[B26] Swann K. Yu Y. (2008). The dynamics of calcium oscillations that activate mammalian eggs. *Int. J. Dev. Biol.* 52 585–594. 10.1387/ijdb.072530ks 18649272

[B27] Tian X. Firsanov D. Zhang Z. Cheng Y. Luo L. Tombline G. (2019). SIRT6 is responsible for more efficient DNA double-strand break repair in long-lived species. *Cell* 177:e622. 10.1016/j.cell.2019.03.043 31002797 PMC6499390

[B28] Toiber D. Erdel F. Bouazoune K. Silberman D. M. Zhong L. Mulligan P. (2013). SIRT6 recruits SNF2H to DNA break sites, preventing genomic instability through chromatin remodeling. *Mol. Cell* 51 454–468. 10.1016/j.molcel.2013.06.018 23911928 PMC3761390

[B29] Vogt E. Kirsch-Volders M. Parry J. Eichenlaub-Ritter U. (2008). Spindle formation, chromosome segregation and the spindle checkpoint in mammalian oocytes and susceptibility to meiotic error. *Mutat. Res.* 651 14–29. 10.1016/j.mrgentox.2007.10.015 18096427

[B30] Watroba M. Dudek I. Skoda M. Stangret A. Rzodkiewicz P. Szukiewicz D. (2017). Sirtuins, epigenetics and longevity. *Ageing Res. Rev.* 40 11–19. 10.1016/j.arr.2017.08.001 28789901

